# Influence of the Encapsulation Efficiency and Size of Liposome on the Oral Bioavailability of Griseofulvin-Loaded Liposomes

**DOI:** 10.3390/pharmaceutics8030025

**Published:** 2016-08-26

**Authors:** Sandy Gim Ming Ong, Long Chiau Ming, Kah Seng Lee, Kah Hay Yuen

**Affiliations:** 1 School of Pharmaceutical Sciences, Universiti of Sains Malaysia, 11800 Penang, Malaysia; khyuen@usm.my; 2 Unit for Medication Outcomes Research and Education (UMORE), Pharmacy, School of Medicine, University of Tasmania, 7001 Hobart, Australia; longchiauming@gmail.com (L.C.M.); kah_seng_81@yahoo.com (K.S.L.); 3 Vector‑borne Diseases Research Group (VERDI), Faculty of Pharmacy, Universiti Teknologi MARA (UiTM), Puncak Alam, 42300 Selangor, Malaysia

**Keywords:** liposomes, oral administration, poorly bioavailable drug, Griseofulvin

## Abstract

The objective of the present study was to investigate the influence of the encapsulation efficiency and size of liposome on the oral bioavailability of griseofulvin-loaded liposomes. Griseofulvin-loaded liposomes with desired characteristics were prepared from pro-liposome using various techniques. To study the effect of encapsulation efficiency, three preparations of griseofulvin, namely, griseofulvin aqueous suspension and two griseofulvin-loaded liposomes with different amounts of griseofulvin encapsulated [i.e., F1 (32%) and F2(98%)], were administered to rats. On the other hand, to study the effect of liposome size, the rats were given three different griseofulvin-loaded liposomes of various sizes, generated via different mechanical dispersion techniques [i.e., FTS (142 nm), MS (357 nm) and NS (813 nm)], but with essentially similar encapsulation efficiencies (about 93%). Results indicated that the extent of bioavailability of griseofulvin was improved 1.7–2.0 times when given in the form of liposomes (F1) compared to griseofulvin suspension. Besides that, there was an approximately two-fold enhancement of the extent of bioavailability following administration of griseofulvin-loaded liposomes with higher encapsulation efficiency (F2), compared to those of F1. Also, the results showed that the extent of bioavailability of liposomal formulations with smaller sizes were higher by approximately three times compared to liposomal formulation of a larger size. Nevertheless, a further size reduction of griseofulvin-loaded liposome (≤400 nm) did not promote the uptake or bioavailability of griseofulvin. In conclusion, high drug encapsulation efficiency and small liposome size could enhance the oral bioavailability of griseofulvin-loaded liposomes and therefore these two parameters deserve careful consideration during formulation.

## 1. Introduction

**Figure 1:**
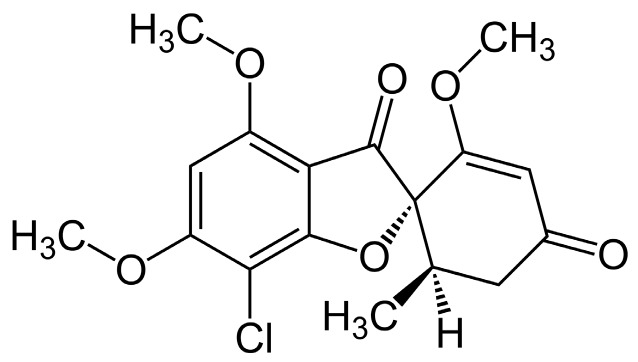
Chemical structure of griseofulvin.

Griseofulvin is an antibiotic fungistatic drug administered orally in the treatment of dermatophyte and ringworm infections. The dosage of griseofulvin varies depending on whether the drug is administered as a microsize or ultramicrosize preparation. In addition, the recommended doses of ultramicrosize griseofulvin vary slightly depending on the manufacturer and the formulation of the drug. It is commercially available as tablets containing 250 or 500 mg microsize or 125, 165, 250 or 330 mg ultramicrosize crystals of griseofulvin, as capsules containing 250 mg microsize griseofulvin and as an oral suspension containing 125 mg/5 mL microsize griseofulvin. It is a white to yellowish-white, crystalline powder with a molecular weight of 352.8. At 20 °C, it is practically insoluble in water, slightly soluble in ethanol, soluble in absolute ethanol (1 in 300), methanol (1 in 250), acetone (1 in 20), chloroform (1 in 25), 1,1,2,2-tetrachloroethane (1 in 3) and freely soluble in dimethylformamide and dimethyl sulfoxide [1,2]. It has a melting point of 217–224 °C and was reported to be thermostable and unaltered by autoclaving. Absorption of griseofulvin from the gastrointestinal (GI) tract is variable and incomplete, but is enhanced by reducing the particle size [3] and co-administration with a fatty meal [4]. Griseofulvin has an elimination half-life of 9–24 h, and is metabolized by the liver mainly to 6-methylgriseofulvin and its glucuronide conjugate which are excreted in the urine [5]. A large dose of griseofulvin (of reduced particle size) appears unchanged in the faeces; less than 1% is excreted unchanged in the urine; some is excreted in sweat. Figure 1 shows the chemical structure of griseofulvin.

The low oral bioavailability of poorly water-soluble drugs poses a great challenge during drug development [6]. Various approaches have been developed to improve the bioavailability by increasing the drug dissolution rate and solubility. Among these approaches, the potential of liposomes as drug delivery systems has been widely acknowledged [7,8,9,10,11] and have shown to enhance the dissolution and absorption of many poorly water-soluble drugs [12,13,14]. Liposomes are naturally occurring self-assembled structures that can also be synthesized in the laboratory [15]. They are composed of one or several lipid bilayers enclosing aqueous compartments and may range in size from tens of nanometers to tens of microns in diameter. They can be formed from a great variety of lipid constituents leading to a wide range of physical properties, thus allowing manipulation of their properties [16]. Lipids forming liposomes may be natural or synthetic. Nevertheless, liposome constituents are not exclusive of lipids as the new generation liposomes, called the polymersomes, can also be prepared from polymers [17]. The physicochemical properties of liposomes such as size, charge, surface properties and encapsulation efficiency can highly influence their in vivo stability and kinetics [18,19]. In addition, these properties can be modified simply by adding new ingredients to the lipid mixture before liposome preparation and/or by variation of preparation methods. For instance, the encapsulation efficiency of hydrophobic drugs could be improved by changing the solvents used for solubilization [20] or the preparation method [21]. In addition, liposomes of various sizes can also be obtained using different sizing techniques such as sonication [22], extrusion [23,24] and homogenization [25,26] and preparation methods [27]. Besides that, the surface charge of liposomes can be altered by addition of charged lipids during formulation [28] or by coating the surface of liposomes with mucoadhesive polymers such as chitosan and carbopol [29,30].

In the present study, the effects of encapsulation efficiency and particle size of liposome on the systemic uptake of griseofulvin were evaluated using rats. Griseofulvin-loaded lipoosmes with different entrapment efficiencies and various sizes were prepared by incorporating different forms of griseofulvin into pro-liposome and using several mechanical methods, respectively.

## 2. Materials and Methods

### 2.1. Materials

Pro-liposome (Pro-lipo duo^®^) was obtained from Lucas Meyer, Champlan, France. The properties and chemical make-up of the pro-liposome are summarized as follows:
Phospholipid typeUnsaturated soybean phosphatidylcholinePhospholipid content50%Hydrophilic mediumGlycerol/ethanolChargeNegativeTransition temperature (Tc)−15 to −30°CConversion temperatureRoom temperature

Griseofulvin was obtained from Sigma-Aldrich (St. Louis, MO, USA). Sodium dihydrogen phosphate dihydrate was purchased from Merck (Damstadt, Germany) while acetonitrile was purchased from Mallinckrodt (Paris, KY, USA). All solvents and chemicals used were of analytical or HPLC grade.

### 2.2. Preparation of Griseofulvin Aqueous Suspension and Griseofulvin-Loaded Liposomes

#### 2.2.1. Griseofulvin Aqueous Suspension

A suspension of griseofulvin (0.25 mg/g) was prepared by dispersing the drug powder in 0.5% (*w*/*v*) Tween 80 aqueous solution. This aqueous suspension was used as a control or reference preparation.

#### 2.2.2. Griseofulvin-Loaded Liposomes

##### Effect of Encapsulation Efficiency

**Formulation 1 (F1)**: Appropriate amount of griseofulvin powder was added to Pro-lipo duo^®^ (2 mg/g pro-liposomes) and mixed thoroughly for 24 h. Then, the mixture was gradually hydrated with purified water. The amount of purified water added was 2 parts to 1 part of the pro-liposome. The mixture was stirred at moderate speed for 30 min at room temperature. Prior to use, the suspension of griseofulvin-loaded liposome produced was further diluted with 5 parts of purified water (relative to the pro-liposome) and stirred for another 5 min to produce a homogenous suspension.

**Formulation 2** **(F2)**: The procedure for preparing F2 is essentially the same as the method used for preparing F1 except that griseofulvin used was dissolved in chloroform. Subsequently, the organic solution containing all materials was dried down by flushing with nitrogen gas, under gentle warming (40 °C) prior to hydration with 2 parts of Milli-Q water to 1 part of the pro-liposomes.

##### Effect of Liposome Size

The liposomal formulations of different sizes were prepared using different hydration techniques, namely, non-shaken, moderate stirring and freeze-thaw sonication. These methods differ in terms of the applied mechanical stress. Initially, a thin film of lipid containing griseofulvin was formed by flushing the organic solution containing all the materials (in a chloroformic solution) with nitrogen gas at 40 °C. The subsequent steps depended on the mechanical dispersion method used and are summarized as follows:

**Non-shaken (NS)**: The dried lipid film was hydrated by addition 7 parts of Milli-Q water (relative to the pro-liposome). Then, the flask was flushed with nitrogen, sealed and allowed to stand for 3 h at room temperature (25 °C). Care was taken not to knock the flask or agitate the medium during the swelling period. After swelling, the vesicles were harvested by swirling the contents of the flasks gently, to produce a milky suspension.

**Moderate stirring (MS)**: The procedure used to prepare this formulation is basically the same as the method used for preparing F2.

**Freeze-thaw sonication (FTS)**: Griseofulvin-loaded liposome produced by MS was transferred into a sealed tube and was rapidly frozen by immersing and shaking the tube in an acetone-dry ice (solid carbon dioxide) bath for 3 min. Then, the frozen liposome was rapidly thawed by immersing and shaking the tube in a water bath (40 °C) for 3 min. Lastly, the tube was placed in the sonicator and its content was sonicated for 5 min at room temperature. Overall, the liposomal formulation was subjected to a total of 10 FTS cycles.

All preparations were prepared extemporaneously and used therein. The preparations produced using the three methods outlined above were designated preparation A, B and C, respectively.

### 2.3. Particle Size Analysis

The liposome size and its distribution was determined by photon correlation spectroscopy (Zetasizer 1000HS, Malvern Instruments Ltd, Worcestershire, UK). Photon correlation spectroscopy, also known as quasi-elastic light scattering, has been mostly preferred because it provides absolute and reproducible results [31]. Prior to measurement, the samples were diluted with Milli-Q water to achieve a count rate of 200–300 K (values displayed by the rate meter). This was done to ensure that the number density of liposomes is low enough to avoid the inter-vesicle interactions [32]. The measurements were made at 25 °C and fixed angle of 90°, where both the effects of reflection and polydispersity are minimized [32]. The “CONTIN” algorithm, which employs matrix techniques to extract a smoothed size distribution [33] was used for the analysis of all samples. The Z average diameter value (ZAve) and polydispersity index which indicate the natural intensity weighted mean and particle size distribution respectively, were recorded and used for comparison of all samples. Standard error mean (SEM) of the average was used to estimate the repeatability of the measurements.

### 2.4. Encapsulation Efficiency Determination

Encapsulation efficiency is an expression of the amount of drug incorporated into the liposome and is normally defined as the percentage of drug bound to liposomes relative the total amount of drug [34]. Determination of this parameter generally requires separation of free drug from the liposomal formulation. Analysis of drug in both the free and encapsulated drug fractions allow calculation of encapsulation efficiency. The encapsulation efficiency of griseofulvin was expressed as the percent of drug encapsulated and calculated using the following formula:

(1) $$\text{Percent encapsulated}=\frac{\left[\text{Total griseofulvin}\right]-\left[\text{Free griseofulvin}\right]}{\left[\text{Total griseofulvin}\right]}\times 100\%$$

#### 2.4.1. Separation of Griseofulvin-Loaded Liposome from Free Griseofulvin

Due to poor solubility of griseofulvin, the free griseofulvin exists in two forms in the external aqueous medium of the liposomal preparation; being the free undissolved and free dissolved form of griseofulvin. The free undissolved form of griseofulvin was separated from the liposomal suspension by centrifugation at 12,800× *g* for 5 min (Eppendorf, Hamburg, Germany). The pellet, which is the free undissolved griseofulvin, was collected and kept at 4 °C until analysis. Meanwhile, the supernatant (consisted of the encapsulated and free dissolved griseofulvin) were filled into polyallomer bell-top Quick-Seal^®^ centrifuge tubes (Beckman, Brea, CA, USA) and heat-sealed using a tabletop sealer before being ultracentrifuged at 215,000× *g* at 20 °C for 2 h (Beckman Optima L-80, Beckman, Brea, CA, USA). The clear supernatant (generated upon ultracentrifugation) which contained the free dissolved griseofulvin was collected and kept at 4 °C until analysis.

#### 2.4.2. Total Griseofulvin

Total griseofulvin refers to the sum of both encapsulated and free griseofulvin that is present in the liposomal preparation. The concentration of total griseofulvin was determined after having dissolved and disrupted liposomal preparation in acetonitrile at 2.5:1 (*v*/*v*) using a vortex mixer for 30 s (Stuart Scientific, Staffordshire, UK), followed by centrifugation for 15 min at 12,800× *g* (Eppendorf, Hamburg, Germany). The clear supernatant which contained the total griseofulvin was then transferred to a new microcentrifuge tube and kept at 4 °C until analysis. All samples were prepared in duplicates.

#### 2.4.3. Determination of the Concentration of Free and Total Griseofulvin

A HPLC method with fluorescence detection [35] was adapted to determine the concentrations of both free and total griseofulvin. The HPLC system consisted of an Agilent 1100 Series Isocratic Pump and Fluorescence Detector (Agilent Technologies, Waldbronn, Germany), a Rheodyne 7725i sample injector valve fitted with a 50 µL sample loop (Rheodyne, Cocati, CA, USA) and a chromatography working station ChemStation Revision A.10.02 (Agilent Technologies). A Genesis C18, 150 mm × 4.6 mm i.d., 4 µm analytical column (Jones Chromatography, Lakewood, CO, USA) was used for the chromatographic separation and was preceded with a refillable guard column (Upchurch Scientific, Oak Harbour, WA, USA) packed with Perisorb RP-18 (30–40 µm, pellicular). The mobile phase comprised 50 mM sodium dihydrogen phosphate and acetonitrile at a ratio of 1:1 *v*/*v*. The system was operated at ambient room temperature (25 °C) with the detector operating at an excitation wavelength of 293 nm and an emission wavelength of 444 nm while the photomultiplier (PMT) was set at a gain of 14. Analyses were run at a flow of 1.0 mL/min and the samples were quantified using peak area.

### 2.5. Animal Experiments

#### 2.5.1. Study Protocol

##### Effect of Encapsulation Efficiency

The study protocol was reviewed and approved by the Animal Ethical Committee, Universiti Sains Malaysia [USM/PPSF/50 (035) Jld. 2, 5 November 2008]. The experiment was carried out using 9 adult male, Sprague-Dawley rats weighing 197–284 g (mean = 247 g, SD = 25 g), according to a 3-period, 3-sequence crossover design, with a one-week washout period between the phases. The rats were randomly divided into three groups of 3 rats each. The rats in each group were administered the preparations according to the sequence shown in Table 1.

The rats were fasted overnight for at least 12 h before administration of the respective preparations by oral intubation. Food was withheld for further 6 h after dosing but the animals had free access to water throughout the study period. For all formulations, the griseofulvin administered dose was 8 mg/kg of the rat body weight. Blood samples of approximately 0.5 mL were collected from the tail vein into heparinized microcentrifuge tubes at 0 (before dosing), 20 min, 40 min, 1, 1.5, 2, 3, 4, 6, 8 and 12 h post-administration. The blood samples were then centrifuged for 15 min at 12,800× *g*. Subsequently, 0.25 mL aliquot of plasma obtained from each blood sample was transferred into a new microcentrifuge tube. All plasma samples were kept at −20 °C until analysis.

##### Effect of Liposome Size

The study protocol was reviewed and approved by the Animal Ethical Committee, University of Science Malaysia [USM/PPSF/50 (036) Jld. 2, 5 November 2008]. The experiment was carried out using 9 adult male, Sprague-Dawley rats weighing 202–350 g (mean = 269 g, SD = 42 g), according to a 3-period, 3-sequence crossover design, with a one-week washout period between the phases. The rats were randomly divided into three groups of 3 rats each. The rats in each group were administered the preparations according to the sequence shown in Table 2.

The rats were fasted overnight for at least 12 h before administration of the respective preparations by oral intubation. Food was withheld for a further 6 h after dosing but the animals had free access to water throughout the study period. For all formulations, the griseofulvin administered dose was 8 mg/kg of the rat body weight. Blood samples of approximately 0.5 mL were collected from the tail vein into heparinized microcentrifuge tubes at 0 (before dosing), 20 min, 40 min, 1, 1.5, 2, 3, 4, 6, 8 and 12 h post-administration. The blood samples were then centrifuged for 15 min at 12,800× *g*. Subsequently, 0.25 mL aliquot of plasma obtained from each blood sample was transferred into a new microcentrifuge tube. All plasma samples were kept at −20 °C until analysis.

#### 2.5.2. Data and Pharmacokinetic Analysis

The preparations were compared using the pharmacokinetic parameters, namely, area under the plasma-concentration curve from time zero to the last sampling time, *t* (AUC_0-t_), maximum plasma concentration (*C*_max_) and time to reach maximum concentration (*T*_max_). The *C*_max_ and *T*_max_ were obtained directly from the plasma concentration versus time data [36], while AUC_0-t_ was calculated using the trapezoidal formula.

#### 2.5.3. Statistical Analysis

Student’s t test was used to compare the ZAve values of F1 and F2. A statistically significant difference was considered at *p* < 0.05. As for the in vivo studies, the AUC_0-t,_ and *C*_max_ values obtained were analyzed statistically using an analysis of variance (ANOVA) procedure appropriate for a three-way crossover study design, that distinguishes effects due to group, subjects/group, period and treatment [37]. AUC_0-t_ and *C*_max_ values were logarithmically transformed prior to analysis. When a significant difference was found, the Tukey’s test was used to determine which of the means differed. On the other hand, the *T*_max_ values of all the preparations were analyzed using the Friedman Test for multiple related samples. A statistically significant difference was considered at *p* < 0.05. In addition, the 90% confidence intervals for the ratio of AUC_0-t,_ and *C*_max_ values of the F1 and F2 over those of the aqueous suspension, and C and B over the A, as well as the ratio of AUC_0-t_, and *C*_max_ values of F2 over those of F1, and C over those of B were calculated using the two one-sided tests procedure [38].

#### 2.5.4. Analysis of Plasma Griseofulvin Concentration

Plasma levels of griseofulvin were determined using a high performance liquid chromatography (HPLC) method described previously (Section 2.4.3). Prior to analysis, a 100 µL aliquot of sample was accurately measured into a microcentrifuge tube (Eppendorf, Hamburg, Germany) and deproteinized by adding 250 µL of acetonitrile. The mixture was then vortex-mixed for 30 s using a vortex mixer (Thermolyne, Dubuque, IA, USA) and centrifuged (Minispin^®^ plus, Eppendorf, Harmburg, Germany) at 12,800× *g* for 10 min. A 50 µL aliquot of the plasma supernatant was injected onto the HPLC system.

## 3. Results

### 3.1. Particle Size of Liposomes

The values of the ZAve and polydispersity index of griseofulvin-loaded liposomes prepared by various techniques are shown in Table 3. It can be inferred from the ZAve values that griseofulvin-loaded liposomes prepared using drug powder (F1) possessed slightly bigger particle sizes than those prepared using a chloroform drug solution (F2). When analyzed using the student’s *t* test, a statistically significant difference (*p* < 0.05) was observed between the ZAve values of the two liposomal preparations. The polydispersity index for both liposomal preparations was found to be closely similar. This indicates that the homogeneity of the size of liposomes produced was not affected by the method of drug incorporated. Nevertheless, both liposomes produced were in the nanometer size range.

Preparation A produced using the NS method possessed the largest particle size among the liposomes prepared using different mechanical dispersion techniques. This was followed by the preparations B and C, prepared using MS and FTS method respectively. Besides that, the polydispersity index for preparation A was also largest among the three preparations. The high polydispersity index value for NS revealed that NS has a very broad particle size distribution. On the other hand, the polydispersity index for both MS and FTS were found to be moderate and similar. This indicates that the FTS method only further reduced the particle size of the liposomes compared to that produced using MS method, but did not affect the homogeneity of the size of liposomes produced. Nonetheless, all three preparations were in the nanometer size range.

### 3.2. Encapsulation Efficiency

Table 4 shows the encapsulation efficiency of griseofulvin-loaded liposomes prepared by various techniques. The encapsulation efficiency of liposomal formulation prepared using a chloroform drug solution (F2) was 3-times higher than that of prepared using drug powder (F1). This implies that the incorporation of griseofulvin into the pro-liposomes was greater when griseofulvin used was in the solution form. Besides that, the mean percentage of griseofulvin encapsulated in preparations A, B and C were essentially similar, being 93.6% ± 0.1%, 92.7% ± 0.4% and 93.2% ± 0.2%, respectively. Thus, the various mechanical dispersion methods have no significant impact on the encapsulation efficiency.

### 3.3. Animal Experiments

**Figure 2:**
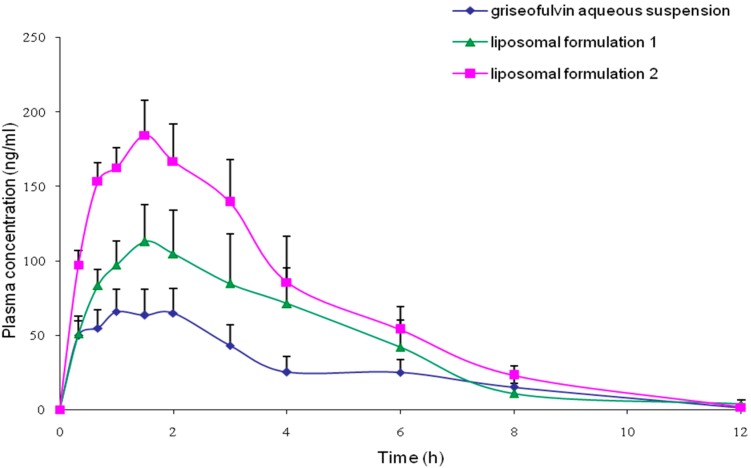
Mean plasma griseofulvin concentration versus time profiles after dosing with griseofulvin aqueous suspension, F1 and F2 (8 mg/kg) (Mean ± SEM, *n* = 9).

The mean plasma griseofulvin concentration versus time profiles obtained with griseofulvin aqueous suspension, F1 and F2 are shown in Figure 2. The profiles show that both liposomal formulations achieved higher plasma drug levels compared to the aqueous suspension, indicating a higher extent of drug absorption attained with liposomal formulations.

Table 5 shows the individual numerical values of AUC_0–t_, *C*_max_ and *T*_max_ obtained after oral administration of the three preparations. It can be seen from the table that mean AUC_0-t_ value of the F1 was almost two times that of griseofulvin aqueous suspension while that of F2 was over two times higher than that of griseofulvin aqueous suspension. When the parameters obtained with the three preparations were analyzed using the ANOVA procedure followed by the post-hoc Tukey’s test, a statistical significant difference (*p* < 0.01) was observed between the AUC_0-t_ values of the liposomal F1 and griseofulvin aqueous suspension, as well as between the values of the F2 and griseofulvin aqueous suspension. Similarly, a statistical significant difference (*p* < 0.05) was also observed between the AUC_0–t_ values of both the liposomal preparations. Thus, it is apparent from the results that the bioavailability of griseofulvin was increased when encapsulated in the liposomes, and that the enhancement was shown to be dependent on drug encapsulation efficiency. In addition, the mean *C*_max_ values of both liposomal formulations were also markedly higher (*p* < 0.05) than that of the griseofulvin aqueous suspension.

The 90% confidence intervals of the AUC_0–t_, and *C*_max_ values of the F2 over those of the other two preparations and F1 over that of griseofulvin aqueous suspension are shown in Table 5. It is apparent from Table 5 that the extent of bioavailability of F2 was about 2.7–3.2 times higher than that of griseofulvin aqueous suspension and 1.5–1.8 times higher compared to that of F1. Whilst, the extent of bioavailability of F1 was about 1.7–2.0 times higher than that of griseofulvin suspension. As for the *C*_max_ values, the 90% confidence intervals showed that the maximum concentration achieved with F2 was 2.4–2.6 times higher than that of the aqueous suspension and 1.5–1.6 times higher than that of F1. Besides that, the 90% confidence interval also showed that the maximum concentration achieved with F1 was 1.6–1.7 times higher than that of the aqueous suspension. In the case of the parameter *T*_max_, no statistical significant difference (*p* > 0.05) was found among the *T*_max_ values of all three preparations. This suggests that the liposomal formulations only increased the extent of bioavailability but not the duration of absorption of griseofulvin compared to the aqueous suspension.

**Figure 3:**
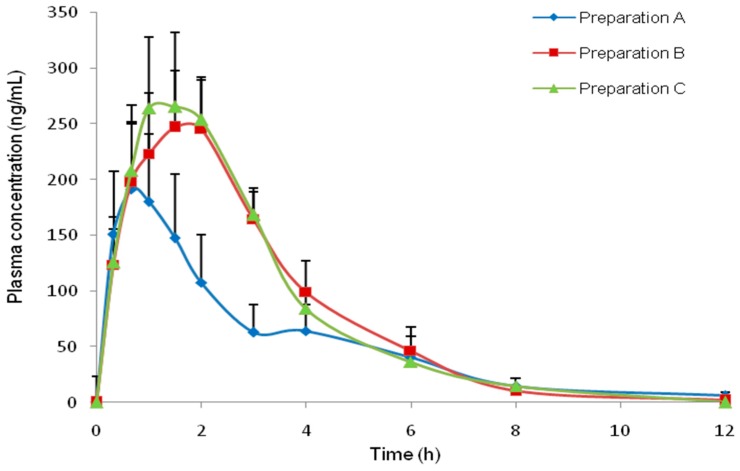
Mean plasma griseofulvin concentration versus time profiles after dosing with liposomal preparations of various particle size (8 mg/kg) (Mean ± SEM, *n* = 9).

The mean plasma griseofulvin concentration versus time profiles of the three preparations of various particle sizes are shown in Figure 3. It is apparent from the plots that the griseofulvin administered as the liposomal preparation of a smaller particle size (preparation B and C produced using MS and FTS method, respectively) showed an enhancement in oral bioavailability compared to the liposomal preparation of larger size (preparation A produced by NS method). Meanwhile, the profiles of both preparations C and B were closely similar although the former achieved a slightly higher peak plasma concentration compared to the latter.

Table 6 shows the individual numerical values of (AUC_0–t_), (*C*_max_) and (*T*_max_) obtained after oral administration of the three preparations. It can be seen from the table that the mean AUC_0–t_ values of both preparations B and C were about two-fold of that of preparation A. When the parameters obtained with the three preparations were analyzed using the ANOVA procedure followed by Tukey’s test, a statistically significant difference (*p* < 0.01) was observed between the AUC_0–t_ values of preparations C and A as well as between the values of preparation B and preparation A. In contrast, no statistically significant difference (*p* > 0.05) was observed between the AUC_0–t_ values of preparations B and C. Thus, it is apparent that the bioavailability of griseofulvin obtained was higher when the drug was encapsulated in liposomes of smaller sizes (<400 nm), compared to that when the size was above 800 nm. The mean *C*_max_ values of both liposomal preparations B and C were also markedly higher (*p* < 0.05) than that of preparation A. However, there was no significant difference between the *C*_max_ values of the former two liposomal preparations, i.e., preparations B and C.

The 90% confidence interval of the ratios of AUC_0–t_, and *C*_max_ values of preparation B over those of preparation A as well as the ratios of liposomal preparation C over those of the other two preparations A and B are shown in Table 6. It is apparent from the table that at 90% confidence level, the extent of bioavailability of preparation B was about 2.6–3.0 times higher than that of preparation A. Whilst, the extent of bioavailability of liposomal preparation C was about 2.9–3.3 times higher than that of preparation A and 1.0–1.2 times higher when compared to that of preparation B at a 90% confidence level. As for the *C*_max_ values, the 90% confidence interval showed that the maximum concentration achieved with the liposomal preparation B was 2.7–2.8 times higher than that of preparation A. Besides that, the 90% confidence interval also showed that the maximum concentration achieved with the liposomal preparation C was 2.9–3.0 times higher than that of preparation A and 1.0–1.1 times compared to that of preparation B. In the case of the parameter *T*_max_, no statistical significant difference (*p* > 0.05) was found among the *T*_max_ values of all three preparations. This suggests that the liposomal preparations of smaller particle sizes (<400 nm, prepared by FTS and MS method) only increase the rate of absorption but not the duration of absorption. In all cases, the duration of absorption was quite short as indicated by the *T*_max_ values.

## 4. Discussion

The oral route has remained the preferred mode of drug administration, mainly due to its convenience and better patient compliance. However, poorly water-soluble drugs suffer low bioavailability when administered orally [39,40]. According to the Biopharmaceutics Classification System (BCS) [41], poorly water-soluble compounds (with solubility less than 100 µg/mL) are classified as either class II or class IV compounds, depending on their intestinal permeability. For BCS class II drugs that exhibit high permeability characteristics, drug absorption is governed by their dissolution in the GI fluids. Whereas, BCS class IV drugs, characterized by both low solubility and poor intestinal wall permeability, are generally poor drug candidates for oral administration. Having low solubility and high permeability, griseofulvin is considered as a Class II drug according to the BCS [42]. Previously, several attempts have been made to enhance griseofulvin bioavailability; by improving both its solubility and dissolution rate. The griseofulvin dissolution rate could be enhanced by nanocrystallization [43,44], micronization [45], complexation with cyclodextrin [46], preparation into nanoparticles from water-dilutable microemulsions [47] and preparation into nanosuspensions from triacetin-in-water emulsion [48]. The use of solid dispersions [49] and bioadhesive polymers also can increase griseofulvin bioavailability [50]. Similarly, griseofulvin solubility increases if solid solutions of griseofulvin are used with polyethylene glycol and sodium dodecyl sulphate [51]. Likewise, a preparation of vinylpyrrolidone/vinylacetate copolymer microspheres with griseofulvin also increases the solubility of griseofulvin [52].

Liposomes are very versatile drug carriers. Drugs with widely varying lipophilicities can be encapsulated into liposomes, either in the phospholipid bilayer, in the encapsulated aqueous volume or at the bilayer interface [53]. Hydrophobic drugs are incorporated into the lipid bilayers, while hydrophilic drugs are usually encapsulated in the aqueous compartments [54]. Liposomes have been used to improve the therapeutic index of various drugs by modifying drug absorption, reducing metabolism, prolonging biological half-life or reducing toxicity. Besides parenteral and topical administration routes, liposomes have been used successfully as an oral drug delivery system [55]. Examples of drugs that showed improved bioavailability when administered orally in liposomal formulations include heparin [56], insulin [57], cyclosporine [58], erythropoietin [59] and cefotaxime [60]. Depending on the desired formulation, different liposome preparation methods can be employed. In the present study, a fusion of proliposomal and solvent evaporation method was utilized to prepare the liposomes. This preparation method was ascertained after several preliminary experiments. One of the previous findings (not included) demonstrated that extended duration of mixing the pro-liposomes and griseofulvin (at room temperature) was insufficient to produce good encapsulation efficiency. Also evident in the present study, the encapsulation efficiency of liposomal preparation F1 (pro-liposomes and griseofulvin mixed for 24 h) was only approximately 30% (Table 4). This could be ascribed to the poor solubility of griseofulvin in aqueous as well as lipid media, which has rendered a slow incorporation of griseofulvin into the pro-liposomes. In addition, elevated temperatures were found to shorten the duration required for mixing to achieve relatively good encapsulation efficiency. For instance, a 3-h mixing at 60 °C yielded encapsulation efficiency of about 30%, similar to that of a 24-h mixing at 25 °C. Such improvement in the encapsulation efficiency could be attributed to enhanced drug solubilization at elevated temperatures. However, prolonged heating above 60 °C caused the pro-liposomes to darken; a sign of phospholipids’ oxidation and degradation [61]. In another experiment, different organic solvents, namely acetone, acetonitrile and chloroform, were used to aid the solubilization and incorporation of griseofulvin into the pro-liposomes. As a variation, griseofulvin was first dissolved in organic solvent prior to addition into the pro-liposomes to ensure complete drug solubilization and homogenous mixing. The mixture was then dried down by flushing with nitrogen gas, under gentle warming (40 °C) before hydration with purified water. As the amount of solvent used was rather small (<1 mL), rotary evaporation was deemed unnecessary for the drying the organic solution formed during the preparation [27]. Results indicated that the use of solvents significantly enhanced the encapsulation efficiency of griseofulvin in the liposomes (over 90% for all types of solvents tested). This is in accord with the findings from the literature that the amount of poorly water soluble drugs encapsulated into liposomes is usually quite high with solvent aid [34] and encapsulation efficiency as high as 100% is attainable, as long as the drugs are not present in quantities which overwhelm the components of the lipid bilayers [17]. Due to limited drug solubility in the external aqueous phase, the solubilized griseofulvin in organic solvents tended to have a higher affinity to the phospholipid membrane components of the liposomes, thereby resulting in enhanced encapsulation efficiency. Among the solvents evaluated, liposomes prepared using chloroform possessed the highest negative zeta potential value and encapsulation efficiency. Therefore, chloroform was considered the most suitable solvent for preparation of griseofulvin-loaded liposomes. Although chloroform is classified as a Class II solvent according to Q3C guidelines [62], the amount used in the present study is very small compared to the conventional solvent evaporation method. A pharmaceutical product is considered safe for use if the amount of residual solvents is below the maximum allowable amounts. According to the Q3C guidelines [62], the maximum allowable amount of chloroform in a product is 60 ppm. Other solvents that are less toxic such as dimethylformamide and dimethyl sulfoxide were not used in the present study due to their very low evaporation rates.

Encapsulation of drug in the liposomes can be important for enhancing the oral bioavailability of the drug. Cefotaxime, for example, showed an increase in the extent of absorption when delivered as a liposomal formulation, compared to cefotaxime aqueous solution or physical mixture [60]. In a different study, Kisel et al. [63] demonstrated that insulin encapsulated liposomes reduced the blood glucose more efficiently than free insulin with empty liposomes. These findings clearly showed that the increase in plasma drug concentration or enhancement in biological activity was attributed to delivering the drug encapsulated in liposomes and not by a mere physical mixture with empty liposomes. Hence, the first part of the present study was aimed to investigate the significance of increased drug encapsulation efficiency in enhancing the systemic uptake of griseofulvin. The study was carried out using Sprague-Dawley rats according to a 3-period, 3-sequence crossover study design to compare the oral bioavailability of griseofulvin from two liposomal formulations of different drug loading against that of an aqueous suspension. The present findings showed that the systemic absorption of griseofulvin given in the form of an aqueous suspension was low and erratic, as evidenced by the high variability in the *C*_max_ and AUC_0–t_ values (Table 5). Other researchers have reported similar large inter-individual difference in the absorption kinetics of griseofulvin [50,64]. The *C*_max_ values ranged from 28.5 to 140.0 ng/mL while the AUC_0-t_ values were between 37.5 and 543.9 h·ng/mL. These values, however, were higher than those reported by Ng [35]. Such discrepancy could be due to the difference in preparation method of aqueous suspension. In the present study, griseofulvin microparticles were dispersed into an aqueous solution with 0.5% (*w*/*v*) Tween 80 aqueous solution. As a result, aggregation of microparticles was reduced, possibly leading to increased bioavailability of micronized griseofulvin. Furthermore, the systemic absorption of griseofulvin was already enhanced when given in the form of F1 (with low encapsulation efficiency), compared to administration as an aqueous suspension. The enhanced absorption or bioavailability could be ascribed to improved dissolution rates and solubilization of griseofulvin during intraluminal processing of the ingested griseofulvin contained in liposomes [40]. Hence, a higher griseofulvin concentration would be available in the GI tract for absorption. Besides that, the rather unique physiology of rats, where bile flow is continuous and independent of food intake [65], would have afforded some limited emulsification of the liposomal formulation, which in turn helped to facilitate absorption of griseofulvin. Although the particle size of the F1 was slightly larger than that of F2, the difference was small and would not impact on the bioavailability of griseofulvin. It is obvious from the present findings that the encapsulation efficiency of griseofulvin-loaded liposomes is crucial for absorption of griseofulvin. There was an approximately two times enhancement of the extent of bioavailability following administration of F2 (higher encapsulation efficiency), compared to F1. According to Fick’s first law, the rate of drug diffusion through the intestinal wall is directly proportional to the concentration gradient between the drug in the gut and the drug in the blood. Therefore, increased drug absorption is expected from a formulation with higher drug dissolution, i.e., F2 in the present study. Other potential mechanisms underlying the enhanced griseofulvin absorption from liposomal formulations include effects on the gastrointestinal membrane permeability and/or lymphatic drug transport, due to the presence of phospholipids. It was reported that liposomal formulation could significantly increase the amount of drug transported by the lymph [60,66,67].

The size of liposome is another important determinant in the effective use of liposome for oral delivery of drugs. Previous studies have demonstrated that the size of liposome affects not only its absorption efficiency in the gastrointestinal tract [68] but also its biodistribution and pharmacokinetic profiles [69]. Based on the results of the in vivo evaluation, the optimized (high encapsulation efficiency) F2, was capable of enhancing the oral bioavailability of griseofulvin by approximately three times, compared to an aqueous suspension of griseofulvin, in adult male, Sprague-Dawley. An alteration of the liposome size of the above formulation might have an impact on the overall oral bioavailability of griseofulvin. Therefore, the aim of the second part of study was to investigate the influence of particle size of liposomes on the enhancement of the systemic uptake of griseofulvin. The study was carried out using nine Sprague-Dawley rats according to a 3-period, 3-sequence crossover study design to compare the oral bioavailability of griseofulvin from three liposomal preparations of different sizes but essentially with similar percentage of drug encapsulated. In the present study, liposomes of different sizes were generated by varying the way of film hydration. The NS method has been known to produce large unilamellar vesicles (LUVs) with high encapsulation volume [27], therefore, it was utilized in this study for preparation of liposomes with large particle size. First reported by Reeves and Dowben [70], this preparation method emphasizes on the swelling procedure. Unlike the widely used hand-shaken method, the hydration and swelling processes were separated into two steps. First, the lipid film was hydrated by flushing with a stream of water-saturated nitrogen. Then, the lipid film was left to swell in an aqueous medium without shaking. In the present study, the NS method was used with a slight modification. The lipid film was hydrated with bulk purified water instead of flushing with water-saturated nitrogen. The swelling procedure, however, remained unchanged. The lipid film was allowed to swell for 3 h at room temperature. The FTS method, on the other hand, was utilized in the present study to produce liposomes with small particle sizes (preparation C). In this method, the freezing and thawing process was aimed at producing physical disruption to the liposomal phospholipid bilayers [27,71] while the sonication process was intended to reduce the size of liposomes. Shaking the preparation in the acetone-dry ice bath was used to achieve rapid freezing of liposomes. Fast freezing rate is essential in order to impose a greater force of stress on liposomes [71] and thus, causing more disruption. In the present study, rapid freezing of liposomes was achieved by shaking the liposomal preparation in the acetone-dry ice bath. During the thawing procedure, a 3-min shaking interval was introduced to ensure complete mixing and to provide ample time for the disrupted bilayer to re-aneal. The preparation was subjected to 10 FTS cycles because the maximum size reduction was recorded after 10 cycles, and after which no further reduction in size was observed. The moderate stirring method was used to produce medium-sized liposomes (preparation B) which served as the control preparation in the present study. The present findings show that the systemic absorption of griseofulvin was lower when the drug was given in the form of larger liposomes (preparation A); compared to administration of the control preparation with moderate-size liposomes (preparation B). The result obtained is in agreement with that reported by Maitani et al. [59], in which the erythropoietin-loaded liposomes of 0.2 µm were absorbed less effectively than those of 0.1 µm diameter. This could possibly be ascribed to smaller particles being more readily taken up by Peyer’s patches than larger particles [72]. Not only were the larger particles less absorbed, they were also found to be more likely to be retained in the epithelial lining of the Peyer’s patches and non-patch microvilli [72,73]. Besides that, a further size reduction (preparation C), however, did not give rise to a further improvement in the bioavailability of griseofulvin, compared to preparation B. The results indicate that at the submicron range of below 400 nm, the liposome uptake or bioavailability is no longer affected by differences in particle size. A similar finding has been earlier reported by Ang [74] in the study of the influence of particle size of nanoparticles on the bioavailability of cefotaxime. Ang [74] found that the uptake of particles was equally efficient as long as the particle sizes were below 500 nm and that the influence of particle size on bioavailability would be clearly evident only when comparing particles in submicron sizes with those in the micron-size range.

## 5. Conclusions

Based on the results obtained from the in vivo studies, liposomal formulation was capable of increasing the bioavailability of griseofulvin. Moreover, the amount of drug encapsulated has a significant impact on the systemic absorption. The larger amount of drug encapsulated was translated into higher oral bioavailability. Also, the size of liposomes affected the extent of griseofulvin bioavailability. Larger liposomes have lower bioavailability compared to the smaller ones. Nevertheless, the uptake or bioavailability of liposomes is equally efficient at the submicron range of below 400 nm, whereby a further size reduction of the liposomes has limited or no impact on the extent of bioavailability. Moreover, the duration of griseofulvin absorption was rather brief and was not influenced by the liposomal sizes.

## Figures and Tables

**Table 1:** Sequence of administration of griseofulvin aqueous suspension, F1 and F2.

Group	Sequence of Administration		
	Phase I	Phase II	Phase III
I	Griseofulvin suspension	F1	F2
II	F2	Griseofulvin suspension	F1
III	F1	F2	Griseofulvin suspension

**Table 2:** Sequence of administration of griseofulvin-loaded liposomes prepared using various methods.

Group	Sequence of Administration		
	Phase I	Phase II	Phase III
I	C	A	B
II	B	C	A
III	A	B	C

**Table 3:** Particle size and size distribution of griseofulvin-loaded liposomes prepared by various techniques (Mean ± SEM, *n* = 18).

Liposomal Formulation	Z ave (nm)	Polydispersity Index
F1	329.9 ± 1.8	0.318 ± 0.009
F2	311.4 ± 2.9	0.292 ± 0.013
A	813.3 ± 5.9	0.984 ± 0.010
B	356.6 ± 5.2	0.497 ± 0.017
C	142.0 ± 1.3	0.551 ± 0.010

**Table 4:** Percentage of griseofulvin encapsulated in liposomal preparations (2 mg/g pro-lipo duo^®^) prepared by various techniques (Mean ± SEM, *n* = 6).

Liposomal Formulation	Percentage of Griseofulvin Encapsulated (%)
F1	31.8 ± 0.9
F2	97.9 ± 0.3
A	93.6 ± 0.1
B	92.7 ± 0.4
C	93.2 ± 0.2

**Table 5:** Individual values of *C*_max_, *T*_max_, AUC_0–t_ and 90% confidence interval for griseofulvin after oral administration of griseofulvin aqueous suspension, F1 and F2.

Rat	Griseofulvin Aqueous Suspension			F1			F2		
	*C*_max_	*T*_max_	AUC_0–t_	*C*_max_	*T*_max_	AUC_0-t_	*C*_max_	*T*_max_	AUC_0–t_
	(ng/mL)	(h)	(h ng/mL)	(ng/mL)	(h)	(h ng/mL)	(ng/mL)	(h)	(h ng/mL)
1	127.5	0.3	220.0	117.9	1.5	290.4	122.5	3.0	556.4
2	118.9	1.0	278.4	160.6	2.0	769.0	177.2	6.0	806.7
3	28.5	0.7	37.5	48.8	0.7	86.4	72.9	2.0	3118.9
4	140.0	2.0	419.2	235.1	1.0	590.4	320.7	1.5	1007.7
5	102.4	3.0	543.9	332.9	3.0	1234.4	426.0	3.0	1451.4
6	113.8	1.0	540.9	186.8	4.0	666.0	410.3	1.5	1113.8
7	69.2	1.0	182.1	89.7	2.0	392.9	226.0	1.5	819.4
8	60.1	6.0	352.0	122.6	1.5	373.5	167.6	0.7	429.8
9	33.1	1.5	94.6	53.9	1.5	267.0	114.5	0.7	380.6
Mean	88.1	1.8	296.5	149.8 ^b^	1.9	518.9 ^a^	226.4 ^b,c^	2.2	1076.1 ^a,c^
SD	41.5	1.8	182.4	91.5	1.0	343.0	130.1	1.7	838.9
CV (%)	47.1	95.6	61.5	61.1	53.9	66.1	57.5	75.1	78.0
CI				1.6–1.7		1.7–2.0	2.4–2.6		2.7–3.2
CI *							1.5–1.6		1.5–1.8

^a^ *p* < 0.01 when compared to aqueous suspension; ^b^ *p* > 0.05 when compared to aqueous suspension; ^c^ *p* < 0.05 when compared to F1; CI is the confidence intervals of the ratio of *C*_max_ and AUC_0–t_ values of F1 and F2 over those of the aqueous suspension; CI * is the confidence intervals of the ratio of *C*_max_ and AUC_0–t_ values of F2 over those of the F1.

**Table 6:** Individual values of *C*_max_, *T*_max_ and AUC_0–t_ and 90% confidence interval for griseofulvin after oral administration of griseofulvin-loaded liposomes prepared using various mechanical dispersion methods.

Rat	Preparation A			Preparation B			Preparation C		
	*C*_max_	*T*_max_	AUC_0–t_	*C*_max_	*T*_max_	AUC_0-t_	*C*_max_	*T*_max_	AUC_0–t_
	(ng/mL)	(h)	(h ng/mL)	(ng/mL)	(h)	(h ng/mL)	(ng/mL)	(h)	(h ng/mL)
1	61.4	0.7	51.1	458.0	0.7	1394.9	331.4	1.5	915.2
2	60.8	1.5	214.6	260.2	0.7	429.2	288.1	2.0	816.9
3	278.5	2.0	1393.3	633.3	0.7	1885.0	724.2	2.0	2169.8
4	30.9	1.0	86.5	45.1	1.5	214.9	173.7	1.0	569.9
5	184.2	2.0	1258.4	283.7	1.0	1140.5	417.1	1.5	1668.8
6	106.2	0.3	277.8	402.1	1.0	847.2	353.8	1.0	763.3
7	318.7	1.0	660.7	414.9	1.5	918.1	212.2	1.0	499.2
8	66.5	4.0	133.9	387.2	1.5	1041.5	152.2	1.0	568.6
9	92.5	4.0	371.0	187.0	0.7	771.0	375.3	1.5	1126.2
Mean	133.3	1.8	494.1	341.3 ^b^	1.0	960.3 ^a^	336.4 ^b^	1.4	1010.9 ^a^
SD	103.6	1.3	506.5	170.1	0.4	496.4	172.1	0.4	564.5
CV (%)	77.7	73.6	102.5	49.9	37.7	51.7	51.2	30.0	55.8
CI				2.7–2.8		2.6–3.0	2.9–3.0		2.9–3.3
CI *							1.0–1.1		1.0–1.2

^a^ *p* < 0.01 when compared to preparation A; ^b^ *p* > 0.05 when compared to preparation A; CI is the confidence intervals of the ratio of *C*_max_ and AUC_0–t_ values of preparation B and preparation C over those of the preparation A; CI * is the confidence intervals of the ratio of *C*_max_ and AUC_0-t_ values of preparation C those of the preparation B.
